# Genomic Microdiversity of *Bifidobacterium pseudocatenulatum* Underlying Differential Strain-Level Responses to Dietary Carbohydrate Intervention

**DOI:** 10.1128/mBio.02348-16

**Published:** 2017-02-14

**Authors:** Guojun Wu, Chenhong Zhang, Huan Wu, Ruirui Wang, Jian Shen, Linghua Wang, Yufeng Zhao, Xiaoyan Pang, Xiaojun Zhang, Liping Zhao, Menghui Zhang

**Affiliations:** State Key Laboratory of Microbial Metabolism, Joint International Research Laboratory of Metabolic and Developmental Sciences, and School of Life Sciences and Biotechnology, Shanghai Jiao Tong University, Shanghai, People’s Republic of China; University of Oklahoma

## Abstract

The genomic basis of the response to dietary intervention of human gut beneficial bacteria remains elusive, which hinders precise manipulation of the microbiota for human health. After receiving a dietary intervention enriched with nondigestible carbohydrates for 105 days, a genetically obese child with Prader-Willi syndrome lost 18.4% of his body weight and showed significant improvement in his bioclinical parameters. We obtained five isolates (C1, C15, C55, C62, and C95) of one of the most abundantly promoted beneficial species, *Bifidobacterium pseudocatenulatum*, from a postintervention fecal sample. Intriguingly, these five *B. pseudocatenulatum* strains showed differential responses during the dietary intervention. Two strains were largely unaffected, while the other three were promoted to different extents by the changes in dietary carbohydrate resources. The differential responses of these strains were consistent with their functional clustering based on the COGs (Clusters of Orthologous Groups), including those involved with the ABC-type sugar transport systems, suggesting that the strain-specific genomic variations may have contributed to the niche adaption. Particularly, *B. pseudocatenulatum* C15, which had the most diverse types and highest gene copy numbers of carbohydrate-active enzymes targeting plant polysaccharides, had the highest abundance after the dietary intervention. These studies show the importance of understanding genomic diversity of specific members of the gut microbiota if precise nutrition approaches are to be realized.

## IMPORTANCE

The manipulation of the gut microbiota via dietary approaches is a promising option for improving human health. Our findings showed differential responses of multiple *B. pseudocatenulatum* strains isolated from the same habitat to the dietary intervention, as well as strain-specific correlations with bioclinical parameters of the host. The comparative genomics revealed a genome-level microdiversity of related functional genes, which may have contributed to these differences. These results highlight the necessity of understanding strain-level differences if precise manipulation of gut microbiota through dietary approaches is to be realized.

## INTRODUCTION

Recent evidence indicates that the dysbiosis of the gut microbiome plays a pivotal role in human diseases such as obesity and diabetes (1, 2). Specific members of the gut microbiota with a causative contribution to disease/health phenotypes can serve not only as a powerful tool for disease diagnosis (3, 4) but also as targets for disease alleviation/treatment through various methods such as drugs (5), fecal microbiota transplantation (6), and diet (7). However, due to the complexity and interindividual variation of the gut microbiota itself and its interactions with the host and diet (8), the precision manipulation of the gut microbiota for achieving optimum human health needs a more rigorous understanding at the genomic and molecular levels.

In one of our previous dietary intervention studies, we found that a diet enriched in nondigestible but fermentable carbohydrates, which was composed of whole grains, traditional Chinese medicinal foods, and prebiotics (the WTP diet), not only significantly changed the gut microbiota but also improved the bioclinical parameters and inflammatory conditions in genetically obese children with Prader-Willi syndrome (9). One particular beneficial bacterium, *Bifidobacterium pseudocatenulatum* was significantly enriched after the dietary intervention, negatively correlated with other potential detrimental species, and positively related to improvement of the clinical parameters of the host (9).

One child from that cohort finished the dietary intervention over 105 days. His bioclinical parameters were improved, with an initial body weight loss of more than 25.8 kg, together with a significant shift of the gut microbial community such as increases of *Faecalibacterium*, *Lactobacillus*, and *Bifidobacterium* spp. Moreover, we found that *B. pseudocatenulatum* was the most dominant *Bifidobacterium* species after the dietary intervention. Intriguingly, we had isolated five strains from his fecal sample on day 105 (10), and these strains had differential responses to the carbohydrate-enriched interventions. To understand the genetic traits involved in the bacterial niche adaption in the gut ecosystem and the interactions with the host and diet (11), we performed comparative genomics on *B. pseudocatenulatum*.

## RESULTS AND DISCUSSION

### Improved bioclinical parameters and shifted gut microbiota.

**FIG 1:**
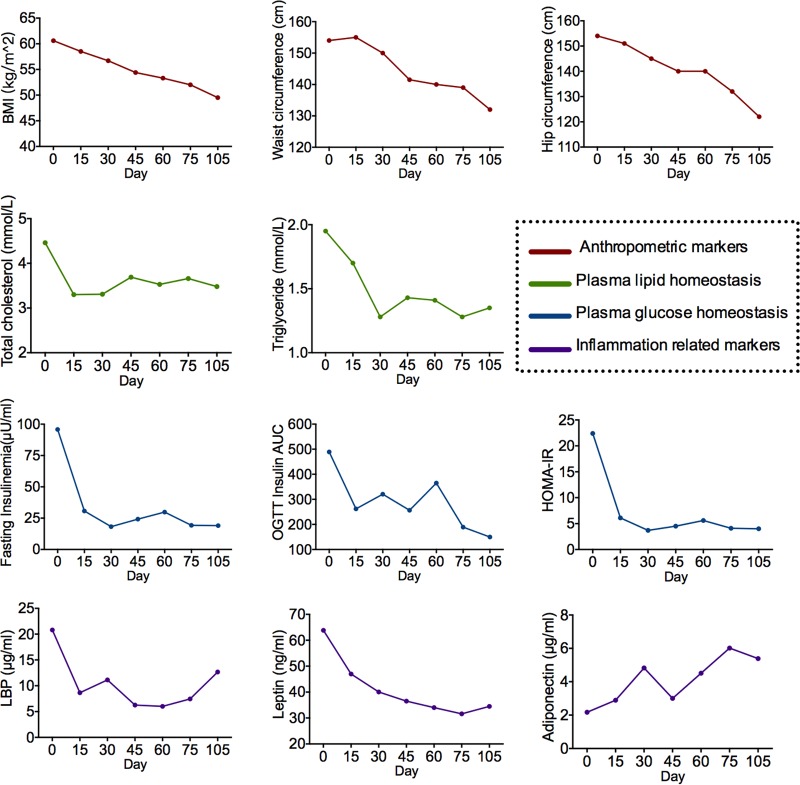
Improved bioclinical parameters and inflammatory conditions after the dietary intervention. Anthropometric markers, plasma lipid homeostasis, plasma glucose homeostasis, and inflammation-related markers are shown. Abbreviations: BMI, body mass index; OGTT, oral glucose tolerance test; LBP, lipopolysaccharide-binding protein; HOMA-IR, homeostatic model assessment of insulin resistance.

The bioclinical variables of the obese child improved during the dietary intervention (Fig. 1; see Fig. S1 in the supplemental material). The child’s body weight was reduced from 140.1 kg to 114.3 kg, and both the plasma glucose and lipid homeostasis were improved to the normal range. Two systemic inflammation markers, C-reactive protein (CRP) and serum amyloid A protein (SAA), were decreased after the dietary intervention. Adiponectin increased from 2.17 μg/ml to 5.39 μg/ml, and leptin decreased from 63.82 ng/ml to 34.47 ng/ml, indicating an alleviation of the “at-risk” phenotype (12). In addition, lipopolysaccharide-binding protein (LBP), a surrogate marker for bacterial antigen load in blood (13), decreased.

10.1128/mBio.02348-16.1 FIG S1  Bioclinical parameters that changed during the dietary intervention. Download FIG S1, PDF file, 0.2 MB. Copyright © 2017 Wu et al. 2017 Wu et al. This content is distributed under the terms of the Creative Commons Attribution 4.0 International license.

**FIG 2:**
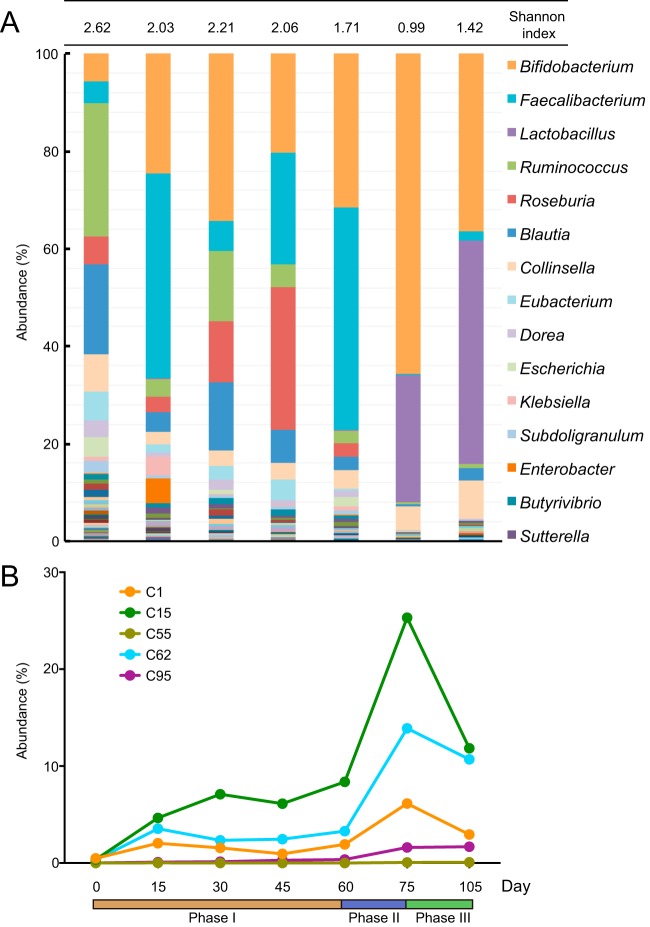
Shifts of the gut microbiota during the dietary intervention. (A) Genus-level gut microbiota compositions at the seven time points. The Shannon index values are shown in panel A. According to the average abundance, the top 15 genera are labeled with their taxonomic names. (B) The differential abundances of the 5 *B. pseudocatenulatum* strains. Phase I, day 0 to day 60, basic dietary intervention; phase II, day 60 to day 75, basic intervention plus 100 g more Formula No. 3; phase III, less Formula No. 1 plus 100 g more Formula No. 3.

We obtained 25.2 × 10^6^ ± 4.8 × 10^6^ (mean ± standard deviation [SD]) high-quality paired-end reads per fecal sample at seven time points (0, 15, 30, 45, 60, 75, and 105 days) via metagenomic sequencing. The composition of the gut microbiota shifted during the dietary intervention (Fig. 2A) and showed different patterns responding to the three interventional phases (phase I, basic intervention from day 0 to day 60; phase II, basic intervention with 100 g more Formula No. 3 from day 60 to day 75; phase III, reduced Formula No. 1 with 100 g more Formula No. 3 from day 75 to day 105 [see Materials and Methods for details]). The defecation frequencies of the obese child in the different phases were similar (on average, three or four times per day), and no diarrhea occurred. The diversity of the gut bacterial community decreased during the dietary intervention, which is consistent with the changes in the cohort (9). At the baseline, *Ruminococcus* and *Blautia* were the two most abundant genera, accounting for 26.95% and 18.41%, respectively. Meanwhile, *Bacteroides* and *Prevotella* were present at low levels, suggesting that the community may belong to enterotype 3 (14). After the dietary intervention, *Ruminococcus* and *Blautia* were reduced to low abundance, while *Bacteroides* and *Prevotella* were almost unaffected by the intervention. Compared with the baseline, *Faecalibacterium*, which was reported to be an anti-inflammatory species (15, 16) and a beneficial (5) commensal bacterium, increased in phase I, with its abundance reaching 41.95% at day 15. The sharp increase in *Faecalibacterium* might contribute to the alleviation of inflammation in the first 15 days, as CRP and SAA decreased by 33.37% and 50.85%, respectively, during this period. *Faecalibacterium* decreased to a low abundance in phases II and III, when more oligosaccharides were provided. On the other hand, *Lactobacillus*, which is well equipped to metabolize oligosaccharides (17), had a low abundance at the baseline and during phase I but became one of the most dominant genera starting from phase II. Several studies have reported the beneficial effects of *Lactobacillus* strains on insulin resistance (18, 19). The dramatic increase of *Lactobacillus* may have contributed to the improvement of insulin sensitivity from phase II as indicated by the remarkable decrease of the oral glucose tolerance test (OGTT) insulin area under the curve (AUC) and steady the OGTT glucose AUC during this period. The abundance of *Bifidobacterium* microorganisms, which have the ability to metabolize a variety of carbohydrates via intra- and extracellular ways (20), was remarkable from day 15 and remained a predominant genus through the entire dietary intervention period: increasing from 27.47% when the child received the basic intervention to 65.53% by day 75, then decreasing to 36.41% when less Formula No. 1 containing complex dietary fibers was provided. These results show that the *Bifidobacterium* population responds to the shifts in carbohydrate resources provided in the three phases, and in each phase, it occupied a superior ecological niche. Given the known beneficial effects of *Bifidobacterium* strains on obesity (21, 22), their essential role in the interaction network of our previous cohort study (9) and their sustained high abundance during the dietary intervention, we speculate that they might have contributed to the continual decreases in body weight, body mass index (BMI), waist circumference, and hip circumference. Hence, we performed a deeper analysis on this genus.

In total, nine *Bifidobacterium* species were identified in the postintervention samples (Fig. S2). Of the nine *Bifidobacterium* species, *B. pseudocatenulatum* was the most dominant, with an abundance of 29.36% in the whole-gut microbiota on day 105. *Bifidobacterium longum*, *B. breve*, and *B. adolescentis* represented 9.94%, 7.61%, and 3.75%, respectively, of the microbiota, and the other five species had abundances of less than 1%.

10.1128/mBio.02348-16.2 FIG S2  Abundance of the nine identified *Bifidobacterium* species in the gut microbial community on day 105. Download FIG S2, PDF file, 0.3 MB. Copyright © 2017 Wu et al. 2017 Wu et al. This content is distributed under the terms of the Creative Commons Attribution 4.0 International license.

### Differential responses of the *B. pseudocatenulatum* strains to the dietary intervention.

For a detailed study of the *B. pseudocatenulatum* population, we isolated and completely sequenced five *B. pseudocatenulatum* strains (strains C1, C15, C55, C62, and C95 [23]) from the fecal sample collected from our subject on day 105 (10). The abundance changes of these five strains at each time point were identified using the Sigma algorithm (24) by aligning the metagenomic data with the complete genomes (Fig. 2B). Before the dietary intervention, all of the strains had low abundances (maximum of 0.5% for *B. pseudocatenulatum* C1). The levels of *B. pseudocatenulatum* C55 and *B. pseudocatenulatum* C95 seemed unresponsive to the dietary interventions, as the former remained low throughout the trial and the latter showed a small increase only during phases II and III. In contrast, the *B. pseudocatenulatum* C1, *B*. *pseudocatenulatum* C62, and *B. pseudocatenulatum* C15 strains were responsive to the dietary interventions. Their changes in abundance were consistent with those observed for the *Bifidobacterium* genus, suggesting that these *B. pseudocatenulatum* strains primarily account for these changes. Indeed, after the dietary intervention, these three strains had increased significantly, especially *B. pseudocatenulatum* C15.

**FIG 3:**
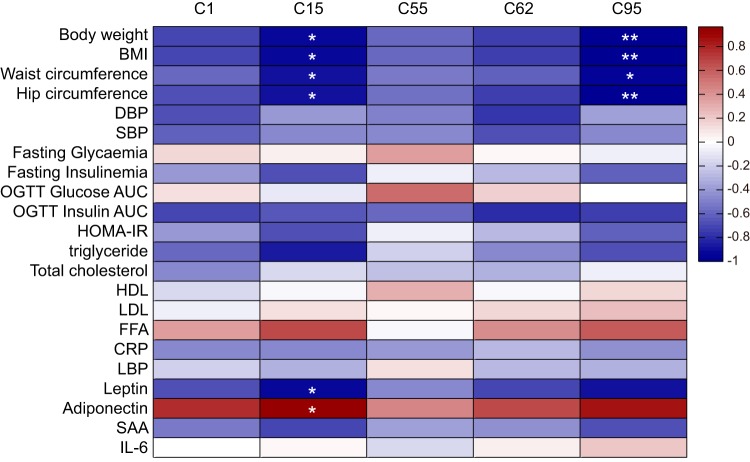
Correlations between the changes in the clinical parameters with the abundance of the five *B. pseudocatenulatum* strains. Spearman correlation was performed. Correlations with an adjusted *P* value of <0.05 (*) or with an adjusted *P* value of <0.01 (**) are indicated by white asterisk(s) (60). Abbreviations: DBP, diastolic blood pressure; SBP, systolic blood pressure; HDL, high-density lipoprotein; LDL, low-density lipoprotein; FFA, free fatty acids; IL-6, interleukin 6.

Consistent with the findings of the previous study (9), the improvements in the bioclinical variables were correlated with the increase in the *B. pseudocatenulatum* strains (Fig. 3). *B. pseudocatenulatum* C95 was negatively correlated with anthropometric markers, including body weight, BMI, waist circumference and hip circumference. Furthermore, *B. pseudocatenulatum* C15 was also correlated with the improvement of inflammatory markers, including the decrease in leptin and the increase in adiponectin.

### Pan-genome analysis of *B. pseudocatenulatum.*

Based on our 5 complete *B. pseudocatenulatum* genomes and the available public data, including 5 draft *B. pseudocatenulatum* genomes and the complete genome of *B. pseudocatenulatum* JCM1200^T^ (25), a total of 11 *B. pseudocatenulatum* genomes were included in the pan-genome analysis. The pan-genome curve showed an asymptotic trend with an average growing rate of 100 genes per genome in the first six iterations and then decreased to a much smaller rate (Fig. S3A). The curve finally arrived at 2,482 genes. This shows that the further incorporation of additional genomes would lead to only a minor increase in the pan-genome size. The core genome curve showed a more evidently asymptotic trend and a clear decrease in the first six iterations (Fig. S3B). The curve finally arrived at 1,427 genes. The pan-genome and core genome trends suggest that *B. pseudocatenulatum* displayed a closed pan-genome, and six genomes are almost sufficient to describe the gene properties of *B. pseudocatenulatum*. On the basis of these results and to avoid the illegibility caused by draft genomes, we used only the six complete genomes in our subsequent analysis to explore the genomic features of *B. pseudocatenulatum*.

10.1128/mBio.02348-16.3 FIG S3  Pan-genome and core genome curves of *B. pseudocatenulatum*. Download FIG S3, PDF file, 0.2 MB. Copyright © 2017 Wu et al. 2017 Wu et al. This content is distributed under the terms of the Creative Commons Attribution 4.0 International license.

### General features of *B. pseudocatenulatum.*

The genomes of our five strains and the *B. pseudocatenulatum* JCM1200^T^ displayed an average of 2,355,185 bp and 56.63 G+C%, which is consistent with the G+C% range of the *Bifidobacterium* genus (26). Five or six rRNA operon loci were identified in the genomes of *B. pseudocatenulatum*, and in each genome, there was one additional copy of the 5S rRNA gene (Table 1). In addition, the heterogeneity across the 16S rRNA genes existed in all the genomes except for that of *B. pseudocatenulatum* JCM1200^T^ (Table S1). On average, 54 tRNA genes were found in each *B. pseudocatenulatum* genome.

10.1128/mBio.02348-16.6 TABLE S1  Heterogeneity of the 16S rRNA genes. Download TABLE S1, DOCX file, 0.1 MB. Copyright © 2017 Wu et al. 2017 Wu et al. This content is distributed under the terms of the Creative Commons Attribution 4.0 International license.

**TABLE 1:** General features of the six complete genomes of *B. pseudocatenulatum*

Feature	Value of feature for strain:					
	C15	C1	C55	C62	C95	JCM1200^T^
Genome length (bp)	2,341,029	2,380,612	2,352,149	2,393,824	2,349,745	2,313,752
No. of genes	1,854	1,883	1,913	1,895	1,868	1,817
No. of tRNA genes	55	54	54	54	54	55
No. of 16S rRNA genes	6	5	6	5	5	6
No. of 23S rRNA genes	6	5	6	5	5	6
No. of 5S rRNA genes	7	6	7	6	6	7
No. of hypothetical proteins	372	381	366	380	363	372
Hypothetical proteins (%)	20	20	19	20	19	20
Gene with assigned function (%)	80	80	81	80	81	80
G+C content (%)	56.59	56.76	56.65	56.78	56.63	56.38
No. of ORFs identified as CAZy	106	104	104	106	106	111

**FIG 4:**
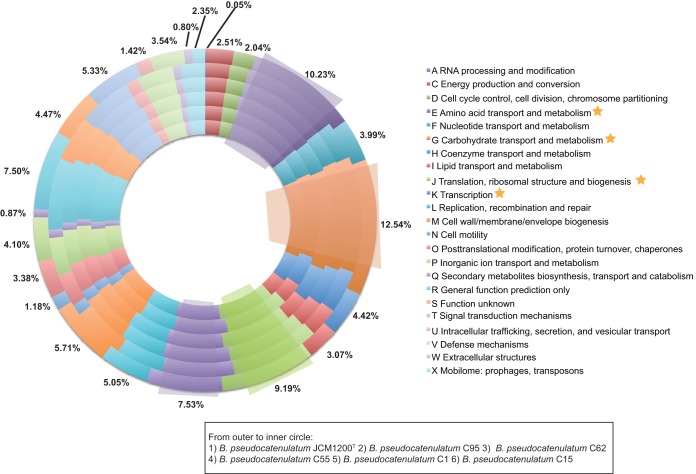
Cluster of Orthologous Groups (COG) classification of the orthologous genes of *B. pseudocatenulatum*. For each COG, the average percentage among the six complete *B. pseudocatenulatum* genomes has been indicated.

An average of 1,871 open reading frames (ORFs) per genome was predicted, with 80% of the detected ORFs with functional assignments via *in silico* prediction based on BLAST against the NCBI nr database, and the remaining 20% were predicted to be hypothetical proteins (Table 1). The identification of orthologous genes according to the COG (Clusters of Orthologous Groups) database (27) showed that the majority of the genes in the genome of *B. pseudocatenulatum* were involved in various housekeeping functions especially those for carbohydrate transport and metabolism (12.54%) as well as amino acid transport and metabolism (10.23%) (Fig. 4). These percentages were in agreement with those of other *Bifidobacterium* genomes (28, 29).

Based on the genomic analysis and supported by experimental evidence, several host colonization factors of bifidobacteria have already been identified, including functions involved in bile resistance and adhesins (11). Resistance to bile is important for the colonization of many intestinal bacteria, as bile acids can have antimicrobial activity at physiological concentrations (30). Bile salt hydrolase and/or bile acid transporter, which confer bile resistance, were identified in all six *B. pseudocatenulatum* strains. In the aspect of adhesion, all of the genomes harbored genes encoding enolase, and DnaK, which has been shown to be a plasminogen-binding related protein in *Bifidobacterium animalis* subsp. *lactis* BI07 (31, 32). Moreover, the genes encoding transaldolase, which is involved in the mucin binding found in four *B. bifidum* strains (33) and von Willebrand factor A, which has been reported to promote adhesion to extracellular matrices (34), existed in each genome as well. The existence of these functional genes suggests that, similar to other *Bifidobacterium* species, *B. pseudocatenulatum* has the genomic basis to colonize in the human intestine.

### Genomic microdiversity of *B. pseudocatenulatum.*

**FIG 5:**
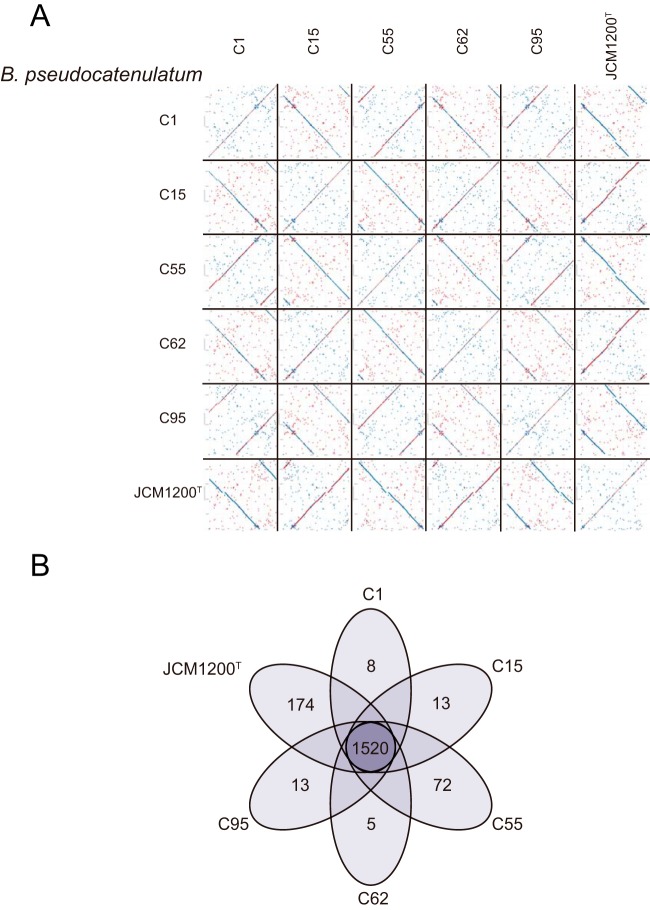
Genomic variations among the *B. pseudocatenulatum* strains. (A) Pairwise dot plot comparisons based on genomic sequences alignment with MUMmer among the six complete *B. pseudocatenulatum* genomes. (B) The Venn diagram shows the number of core and unique gene groups in each strain.

The average nucleotide identity (ANI) among the six complete *B. pseudocatenulatum* genomes met the threshold for species demarcation (35), as the minimum value was 97.76%. The similarity within the strains isolated from the same habitat (*B. pseudocatenulatum* C1, C15, C55, C62, and C95, minimum ANI of 99.88%) was slightly higher that the similarity of the five strains to *B. pseudocatenulatum* JCM1200^T^ (maximum ANI of 97.80%), which was isolated from a different habitat. These results indicated the high degree of synteny across all these genomes, which is confirmed by the dot plot alignments for the genomes (Fig. 5A), although less colinearity and some differences (including indels) were apparent in the dot plots between *B. pseudocatenulatum* JCM1200^T^ and the other five strains. A notable example of this variation was the *eps* gene cluster, which encodes exopolysaccharides (EPSs). EPS can form a slime layer that is attached to the cell and also can be released into the environment (36). Some EPSs produced by *Bifidobacterium* were considered to potentially contribute several beneficial activities to their hosts, including the modulation of the immune system, antagonism against pathogens, functions as scavenging agents, and modulation of the microbial community (37). One copy of the *eps* gene cluster was identified in each complete genome of *B. pseudocatenulatum* (Fig. S4), and these *eps* clusters were identical in our five *B. pseudocatenulatum* strains but quite different from that found in *B. pseudocatenulatum* JCM1200^T^.

10.1128/mBio.02348-16.4 FIG S4  Physical maps of the predicted *eps* clusters from the six complete *B. pseudocatenulatum* genomes. Download FIG S4, PDF file, 0.2 MB. Copyright © 2017 Wu et al. 2017 Wu et al. This content is distributed under the terms of the Creative Commons Attribution 4.0 International license.

All ORFs identified on the six complete genomes were compared with BLASTP and further clustered with the Markov cluster (MCL) algorithm, which revealed the presence of 2,115 gene groups (Fig. 5B). Of these gene groups, ~72% (1,520) were shared by all six *B. pseudocatenulatum* genomes, which represent the core genome of* B. pseudocatenulatum*. A total of 312 dispensable gene groups, which were present only in a subset of the examined *B. pseudocatenulatum* genomes, were identified. More than 61.48% of the unique groups were specific to *B. pseudocatenulatum* JCM1200^T^. Similar results were obtained based on COG assignments, with 1,101 COG families identified from the six complete *B. pseudocatenulatum* genomes. Of these COG families, 59 COG families were present only in a subset of the examined *B. pseudocatenulatum* genomes, and 37 additional COGs were unique to a single strain, with 35 of these identified only in *B. pseudocatenulatum* JCM1200^T^ (Fig. S5). There were also 46 COG families that were found in our isolates that were absent from the *B. pseudocatenulatum* JCM1200^T^ genome. Interestingly, at least some of these differences relate to the mobilome and DNA rearrangement, with our isolates possessing a larger number of unique COGs related to prophage functions, cellular processes, and signaling, while the *B. pseudocatenulatum* JCM1200^T^ possessed a larger number of unique COGs, including those involved with the clustered regularly interspaced short palindromic repeat (CRISPR)/Cas system(s).

10.1128/mBio.02348-16.5 FIG S5  Distribution of dispensable and unique COGs in the six *B. pseudocatenulatum* genomes. Download FIG S5, PDF file, 0.6 MB. Copyright © 2017 Wu et al. 2017 Wu et al. This content is distributed under the terms of the Creative Commons Attribution 4.0 International license.

Particularly, our isolates and *B. pseudocatenulatum* JCM1200^T^ were exposed to distinct carbohydrate resources. The former were isolated from the fecal sample of the postintervention child, who received mixed materials from whole grains and traditional Chinese medicine (TCM) food plants that are rich in dietary fiber and powders, including fructo-oligosaccharides and oligoisomaltoses, while the latter was isolated from feces from an infant, who was speculated to receive relatively simpler carbohydrates. Correspondingly, genetic variations involved in carbohydrate transport and metabolism were found; for instance, *B. pseudocatenulatum* JCM1200^T^ lacked COG0383 (alpha-mannosidase), COG3594 (fucose 4-*O*-acetylase or related acetyltransferase), COG4209 (ABC-type polysaccharide transport system, permease component), and COG4214 (ABC-type xylose transport system, permease component) but uniquely had COG1554 (trehalose and maltose hydrolase [possible phosphorylase]).

**FIG 6:**
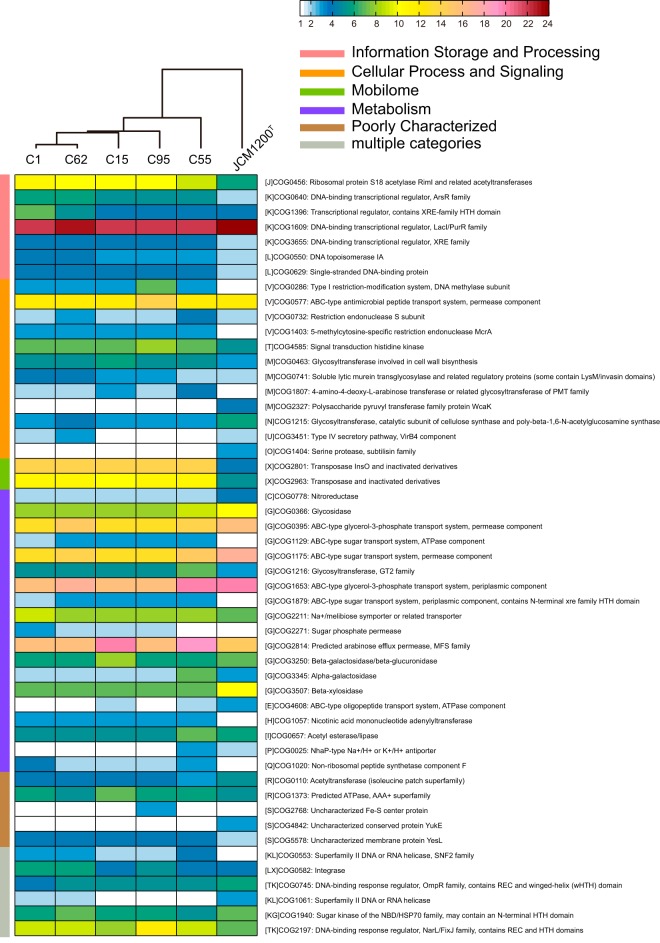
Distribution of core COGs having a disparity in copy number with at least two copies. The heatmap shows the copy number of genes annotated as the particular COG function. The strains are clustered with the Euclidean distance and the Ward linkage method.

The different copy numbers of the core COGs also contributed to the microdiversity of *B. pseudocatenulatum*. Among the 1,005 core COGs that appeared in each complete *B. pseudocatenulatum* genome, 51 COGs involved in various functional categories had a disparity in the copy number with at least two copies (Fig. 6). According to the distribution of these COGs, *B. pseudocatenulatum* JCM1200^T^ was the most different strain compared to our five strains.

Previous studies have reported the microdiversity of different strains in the same species originating from the same habitat (38–40). Among our five *B. pseudocatenulatum* strains, the differences appeared to result from variations in the copy numbers of genes assigned to specific core COG families, instead of differences in terms of the presence/absence of unique and/or dispensable COGs. The clustering result (Fig. 6) was consistent with the differential responses of the strains to the carbohydrate interventions. In that context, *B. pseudocatenulatum* C95 and *B. pseudocatenulatum* C55, which were largely unaffected by the dietary intervention, were clearly separated from the three responsive strains. *B. pseudocatenulatum* C1 and *B. pseudocatenulatum* C62, which had moderate responses to the dietary intervention, grouped together. *B. pseudocatenulatum* C15, the strain that was the most responsive to the dietary intervention, was further separable from *B. pseudocatenulatum* C1 and *B. pseudocatenulatum* C62.

With respect to carbohydrate transport and metabolism, *B. pseudocatenulatum* C15 had the highest copy number of COG2814 (predicted arabinose efflux permease, major facilitator superfamily [MFS]) and COG3250 (beta-galactosidase/beta-glucuronidase). Moreover, among the five strains, 106 ± 2 (mean ± SD) ORFs were identified as carbohydrate-active enzyme (CAZy) genes, which accounted for 41 CAZy families. The *B. pseudocatenulatum* C15 genome harbored all of the identified CAZy families and had the greatest copy numbers of these genes. It also harbored the highest copy numbers of genes encoding carbohydrate esterases, which are reported to deacetylate plant polysaccharides to overcome the complexity and cooperate with glycoside hydrolases in plant polysaccharide degradation (41). These features are presumed to be the principal differences accounting for the responsiveness of *B. pseudocatenulatum* C15 to the dietary intervention, whereby it accounts for more than 50% of the total *B. pseudocatenulatum*, and a substantial proportion of the entire bifidobacterial population throughout the dietary intervention.

### Conclusions.

In a previous study investigating how the WTP diet results in weight loss in genetically obese persons, *B. pseudocatenulatum* was identified as the most abundant *Bifidobacterium* species. Here, we show that specific strains of *B. pseudocatenulatum* show variations in their response to the dietary intervention, and we use comparative genomics to identify possible reasons underlying these dynamics. The five *B. pseudocatenulatum* strains isolated as part of this study showed some differences relative to *B. pseudocatenulatum* JCM1200^T^, which was isolated from feces from an infant, which indicates the effect of different environmental parameters on genome microdiversity (42). Much of the observed microdiversity among our five isolates are the variations in the copy numbers of core COG families, and these differences provide a plausible explanation for the variations in the populations of the five strains during the dietary intervention. In particular, *B. pseudocatenulatum* C15, which genetically had the more diverse and greater copy numbers of CAZy genes for plant polysaccharides, had the greatest abundance in response to the dietary intervention. The coexistence and distribution of multiple strains are intuitive because it would support the survival of the population as a whole over a broader range of environmental conditions than would be possible for a homogeneous population (43). Thus, the coexistence of the five strains with diverse responses to the dietary intervention may work as a mechanism to ensure the stability and restoration of important beneficial species such as *B. pseudocatenulatum* in human gut. Importantly though, the five *B. pseudocatenulatum* strains identified in this study were also found to have different correlations with bioclinical parameters of the host, suggesting that at least some of the beneficial functions ascribed to changes in the gut microbiota are strain specific (44). More studies of the type presented here will be necessary to ensure that the full potential of the health benefits sought from dietary manipulations of the gut microbiota are realized.

## MATERIALS AND METHODS

### Clinical investigation.

The study was performed with the approval of the Ethics Committee of the School of Life Sciences and Biotechnology, Shanghai Jiao Tong University (2012-2016). The clinical trial was registered at the Chinese Clinical Trial Registry (ChiCTR-ONC-12002646). Written informed consent was obtained from the guardians of the obese child.

In Guangdong Women and Children Hospital, Guangzhou, China, the obese child received a dietary intervention for 105 days in the hospital. The volunteer did not take part in any exercise program. A diet based on whole grains, traditional Chinese medicinal foods, and prebiotics (WTP diet) (7) was administered in combination with appropriate amounts of vegetables, fruits, and nuts according to a dietician’s advice. The three ready-to-consume preprepared foods, Formula No. 1, Formula No. 2, and Formula No. 3, in the diet were manufactured by Perfect (China) Co., Ltd. The intervention was divided into three phases. In phase I (day 0 to day 60), the child received the basic diet (9). In phase II (day 60 to day 75), he consumed 100 g more Formula No. 3. During phase III (day 75 to day 105), he still consumed 100 g more Formula No. 3, but less Formula No. 1 was provided.

Biological samples, anthropometric data, and clinical laboratory analysis were obtained at the seven time points (0, 15, 30, 45, 60, 75, and 105 days). The bioclinical parameters were measured as in our previous study (9).

### Metagenomic sequencing and analysis.

DNA extraction from fecal samples for metagenomic sequencing was conducted as previously described (45). Seven samples were sequenced using the Illumina HiSeq 2000 platform at Shanghai Genergy Biotechnology Co., Ltd. DNA libraries were prepared following Illumina’s instructions. Cluster generation, template hybridization, isothermal amplification, linearization, blocking, and denaturing and hybridization of the sequencing primers were performed according to the workflow indicated by the provider. Libraries were constructed followed by high-throughput sequencing to obtain paired-end reads with 151 bp in the forward and reverse directions.

Flexbar (46) was used to trim the adapter from the reads. Prinseq (47) was employed (i) to trim the reads from the 3′ end until reaching the first nucleotide with a quality threshold of 20, (ii) to remove read pairs if either read was shorter than 75 bp or contained “N” bases, and (iii) to deduplicate the reads. Reads that could be aligned to the human genome (*Homo sapiens*, UCSC hg19) were removed (aligned with Bowtie 2 [48], using –reorder–no-hd–no-contain–dovetail). On average, 25.3 × 10^6^ ± 4.1 × 10^6^ (mean ± SD) paired-end reads for each sample were retained and used for further analysis.

MetaPhlan (49) (–bt2_ps very-sensitive-local) was used to calculate the abundance of *Bifidobacterium* species on day 105. Sigma (24) was used to calculate the abundance of our five *B. pseudocatenulatum* strains.

### Whole-genome sequencing and data assembly.

The genomes of *B. pseudocatenulatum* C1, *B*. *pseudocatenulatum* C15, *B*. *pseudocatenulatum* C55, *B*. *pseudocatenulatum* C62, and *B. pseudocatenulatum* C95 were sequenced on a PacBio RS II sequencing instrument with approximately 245-, 415-, 285-, 459-, and 198-fold coverage, respectively (Nextomics Biosciences, Wuhan, China). HGAP/Quiver (50) was used to *de novo* assemble the subreads, followed by Minimus2 (51) and Quiver. The genome of each strain was assembled into one contig that corresponded to its chromosome.

### General feature prediction.

Open reading frame (ORF) prediction was performed by the combination of Prodigal v2_60 (52) and BLAST alignment. The identified ORFs were then annotated using BLASTP against the NCBI nr database. rRNA genes were detected using RNAmmer v1.2 (53), and tRNA genes were identified with tRNAscan-SE v.14 (54). The identity matrix between all 16S rRNA gene was calculated with USEARCH v8.0.1517 (55). The assignment of COGs was done with COGtriangles (ftp://ftp.ncbi.nih.gov/pub/wolf/COGs/COGsoft/).

### Pan-genome calculation.

PGAPv1.12 (56) was used to calculate the pan-genome of *B. pseudocatenulatum*. Functional gene clustering was performed by the GF (gene family) method, and the pan-genome profile was then built.

### Genomic comparison.

Software package MUMmer v3.0 (57) was used to perform the whole-genome sequence alignments at the nucleotide level. For each genome pair, the average nucleotide identity (ANI) was calculated using the program JSpecies version 1.2.1 (35). At the protein level, all sequences were pairwisely compared using BLASTP (maximum E value of 1e−10, minimum alignment identity of 50%, and minimum alignment coverage of 50% for either protein) and then clustering into gene families using the Markov cluster (MCL) algorithm implemented in PGAP v1.12.

### Identification of exopolysaccharide (EPS) gene cluster.

*In silico* analysis of the bifidobacterial *eps* cluster was performed by searching for the putative priming-GTF (p-gtf) gene, *rfbP* (GenBank accession no. NP_695444) and *cpsD* (accession no. NP_695447), in each genome (37), followed by manually checking the genes surrounding the p-gtf gene.

### Identification of Carbohydrate-Activated Enzymes (CAZys).

A local-version database of dbCAN v3.0 (58) was downloaded. Genes in each genome were aligned to the database using HMMscan (59). The alignment was parsed with hmmscan-parser.sh provided by dbCAN, and the best hit was retained.

### Availability of data and materials.

All the genomes generated in this study and the metagenomic data set have been deposited in the European Nucleotide Archive (ENA) (http://www.ebi.ac.uk/ena) under accession number PRJEB18557. All the other genomes of *B. pseudocatenulatum* used for our analysis were downloaded from the NCBI database with the following GenBank accession numbers: AP012330.1, CDPW00000000.1, ABXX02000001, JEOD01000001, and JGZF01000001. The *B. pseudocatenulatum* D2CA genome was downloaded from MetaHIT (http://www.sanger.ac.uk/resources/downloads/bacteria/metahit/).
