# Supplementary material for: Genomic Microdiversity of Bifidobacterium pseudocatenulatum Underlying Differential Strain-Level Responses to Dietary Carbohydrate Intervention
Source: mBio. 2017 Feb 14;8(1):e02348-16. doi: 10.1128/mBio.02348-16 (PMC5312088; doi:10.1128/mBio.02348-16)
Supplement: FIG S1 [file mbo001173185sf1.pdf]

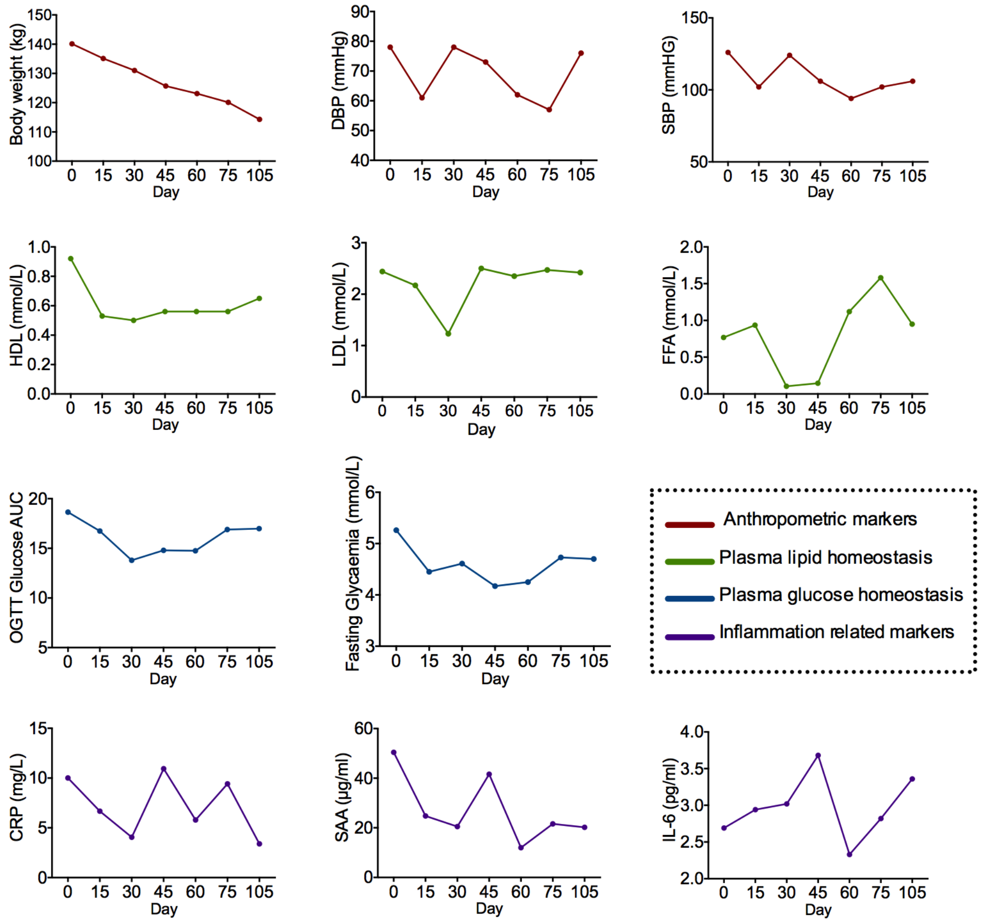

Figure S1 The bioclincial parameters changed during the intervention. (red) Anthropometric markers. (green) Plasma lipid homeostasis. (blue) Plasma glucose homeostasis. (purple) Inflammation related markers. SAA: serum amyloid A protein; CRP: C-reactive protein
