# Supplementary material for: Genomic Microdiversity of Bifidobacterium pseudocatenulatum Underlying Differential Strain-Level Responses to Dietary Carbohydrate Intervention
Source: mBio. 2017 Feb 14;8(1):e02348-16. doi: 10.1128/mBio.02348-16 (PMC5312088; doi:10.1128/mBio.02348-16)
Supplement: FIG S2 [file mbo001173185sf2.pdf]

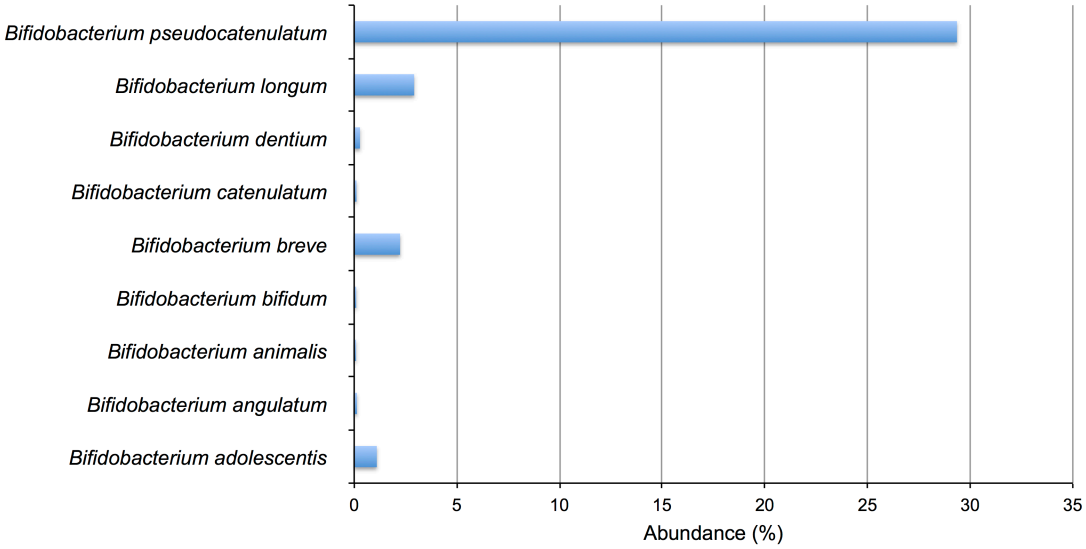

Figure S2 The abundance of the 9 identified *Bifidobacterium* species in the gut microbial community on Day 105.
