# Supplementary material for: Genomic Microdiversity of Bifidobacterium pseudocatenulatum Underlying Differential Strain-Level Responses to Dietary Carbohydrate Intervention
Source: mBio. 2017 Feb 14;8(1):e02348-16. doi: 10.1128/mBio.02348-16 (PMC5312088; doi:10.1128/mBio.02348-16)
Supplement: FIG S3 [file mbo001173185sf3.pdf]

A

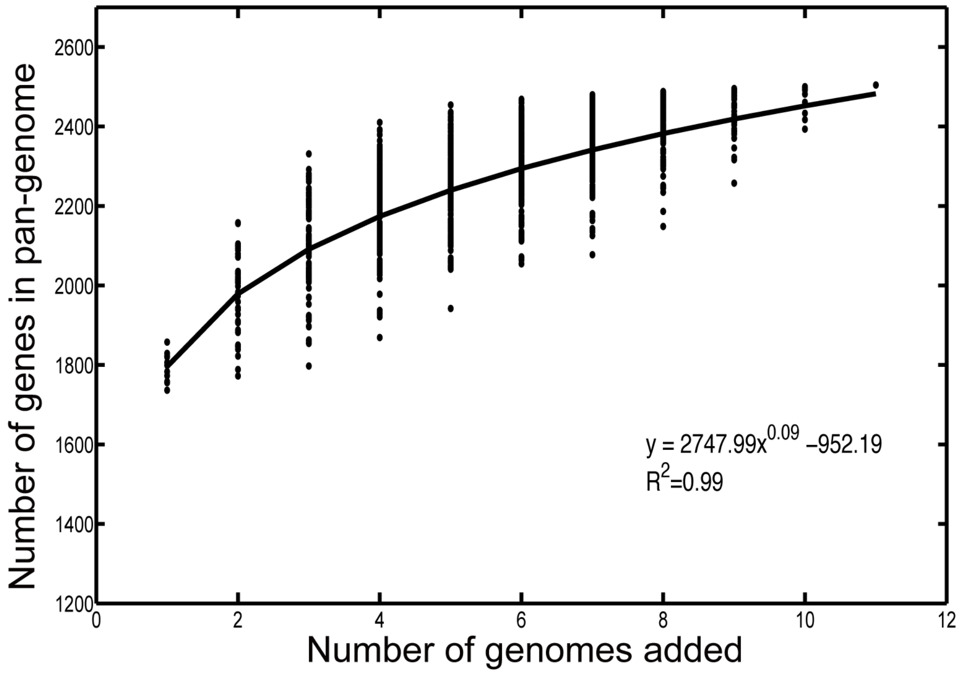

B

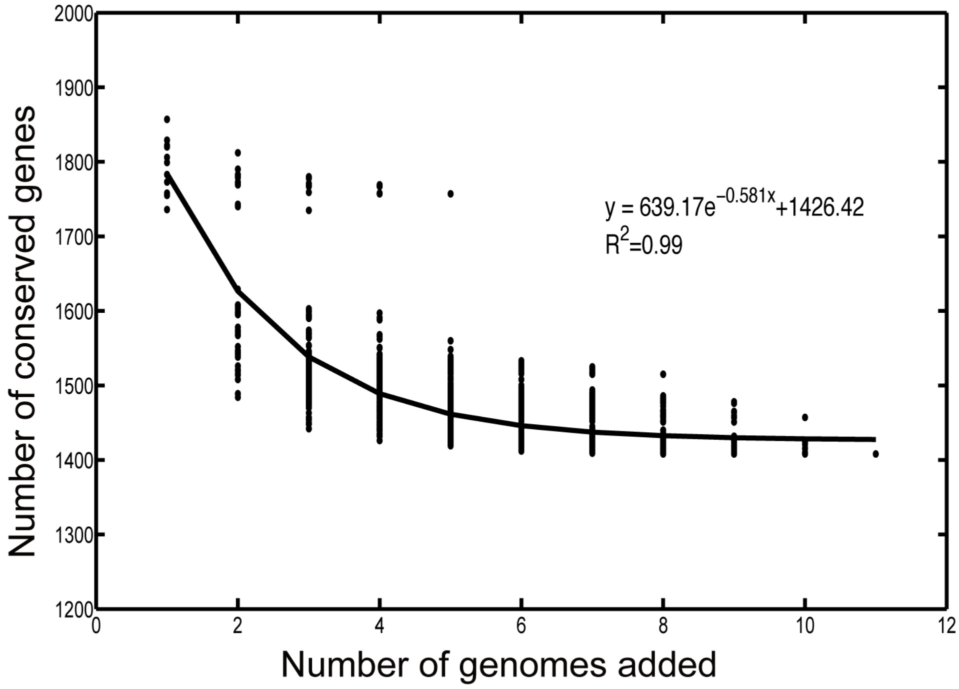

Figure S3 Pan-genome and core-genome curve of B.pseudocatenulatum. (A) Accumulated number of genes in the B.pseudocatenulatum pan-genome plotted against the number of genomes added. The deduced mathematical function is also indicated. (B) The cumulative decreased number of genes attributed to the core-genome plotted against the number of added genomes. The deduced mathematical function is also reported
