# Supplementary material for: Genomic Microdiversity of Bifidobacterium pseudocatenulatum Underlying Differential Strain-Level Responses to Dietary Carbohydrate Intervention
Source: mBio. 2017 Feb 14;8(1):e02348-16. doi: 10.1128/mBio.02348-16 (PMC5312088; doi:10.1128/mBio.02348-16)
Supplement: FIG S4 [file mbo001173185sf4.pdf]

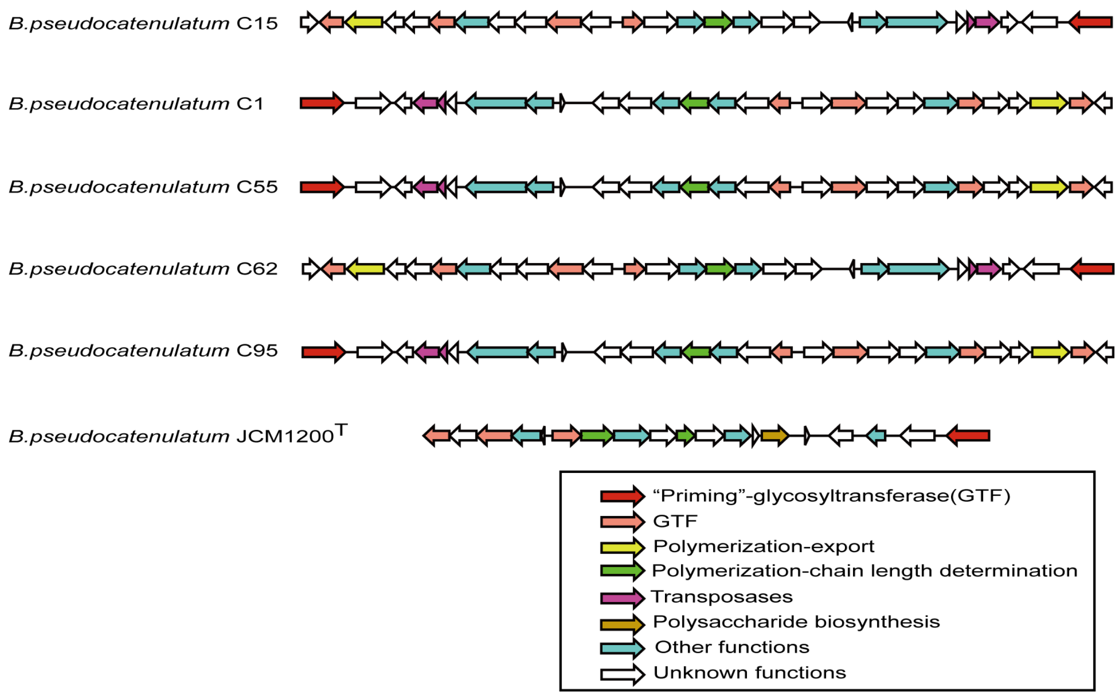

Figure S4 Physical maps of the predicted *eps* clusters from the six complete *B.pseudocatenulatum*. The genes were showed as colored arrows according to their potential functions.
