# Supplementary material for: Genomic Microdiversity of Bifidobacterium pseudocatenulatum Underlying Differential Strain-Level Responses to Dietary Carbohydrate Intervention
Source: mBio. 2017 Feb 14;8(1):e02348-16. doi: 10.1128/mBio.02348-16 (PMC5312088; doi:10.1128/mBio.02348-16)
Supplement: FIG S5 [file mbo001173185sf5.pdf]

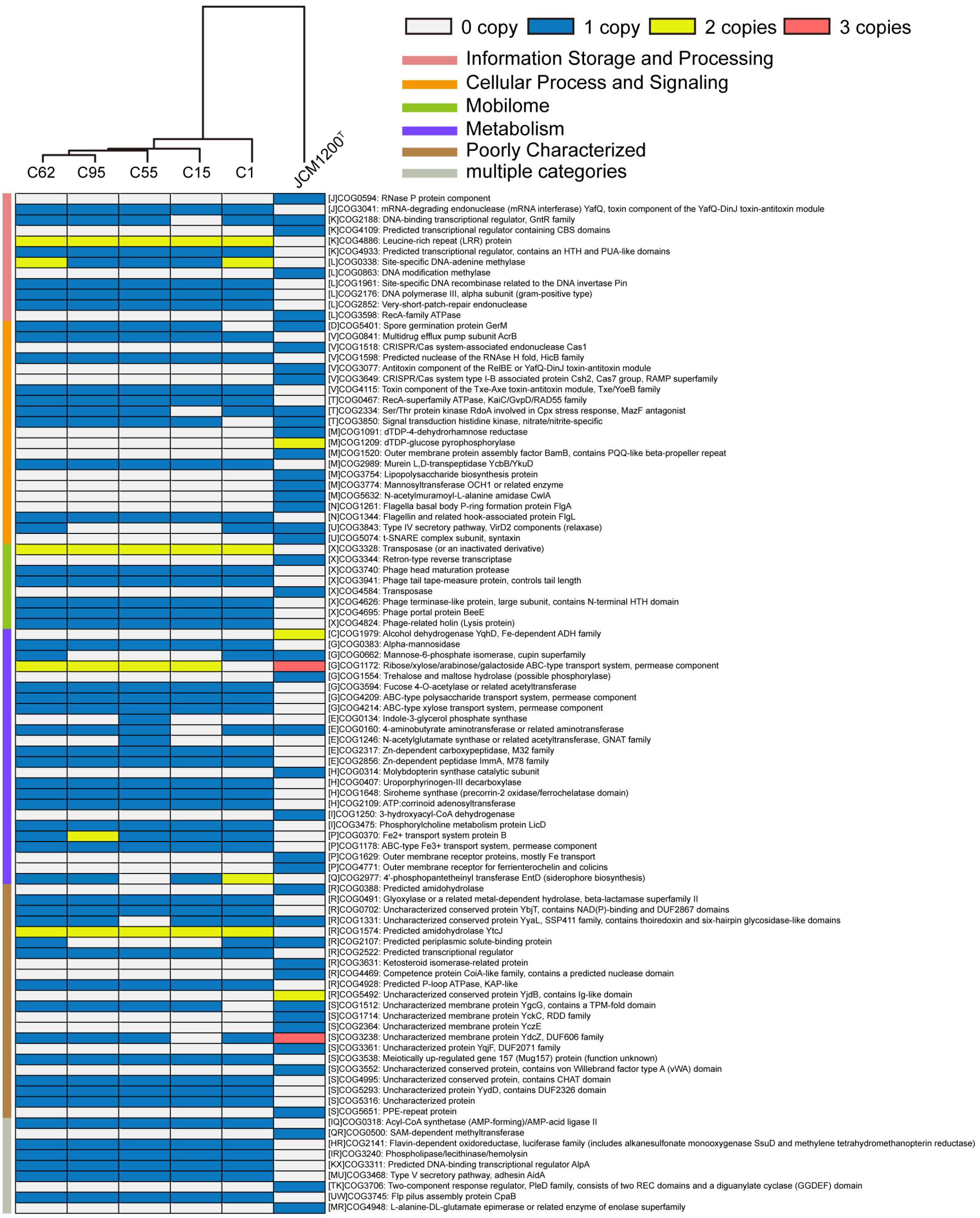

Figure S5 Distribution of dispensable and unique COGs in the six *B.pseudocatenulatum* genomes. The heatmap shows the copy number of genes annotated as the particular COG function. The strains are clustered with Jaccard distance and Ward linkage method.
