# Supplementary material for: Genomic Microdiversity of Bifidobacterium pseudocatenulatum Underlying Differential Strain-Level Responses to Dietary Carbohydrate Intervention
Source: mBio. 2017 Feb 14;8(1):e02348-16. doi: 10.1128/mBio.02348-16 (PMC5312088; doi:10.1128/mBio.02348-16)
Supplement: Supplementary file 6 [file mbo001173185st1.docx]

Table S1. The heterogeneity of 16S rRNA gene

B.pseudocatenulatum C1						
	Copy1	Copy2	Copy3	Copy4	Copy5	
Copy1	100	100	100	100	99.9	
Copy2	100	100	100	100	99.9	
Copy3	100	100	100	100	99.9	
Copy4	100	100	100	100	99.9	
Copy5	99.9	99.9	99.9	99.9	100	
B.pseudocatenulatum C15						
	Copy1	Copy2	Copy3	Copy4	Copy5	Copy6
Copy1	100	100	100	99.9	100	99.7
Copy2	100	100	100	99.9	100	99.7
Copy3	100	100	100	99.9	100	99.7
Copy4	99.9	99.9	99.9	100	99.9	99.6
Copy5	100	100	100	99.9	100	99.7
Copy6	99.7	99.7	99.7	99.6	99.7	100
B.pseudocatenulatum C55						
	Copy1	Copy2	Copy3	Copy4	Copy5	Copy6
Copy1	100	100	100	100	100	99.9
Copy2	100	100	100	100	100	99.9
Copy3	100	100	100	100	100	99.9
Copy4	100	100	100	100	100	99.9
Copy5	100	100	100	100	100	99.9
Copy6	99.9	99.9	99.9	99.9	99.9	100
B.pseudocatenulatum C62						
	Copy1	Copy2	Copy3	Copy4	Copy5	
Copy1	100	100	99.9	100	100	
Copy2	100	100	99.9	100	100	
Copy3	99.9	99.9	100	99.9	99.9	
Copy4	100	100	99.9	100	100	
Copy5	100	100	99.9	100	100	
B.pseudocatenulatum C95						
	Copy1	Copy2	Copy3	Copy4	Copy5	
Copy1	100	99.9	100	100	100	
Copy2	99.9	100	99.9	99.9	99.9	
Copy3	100	99.9	100	100	100	
Copy4	100	99.9	100	100	100	
Copy5	100	99.9	100	100	100	
B.pseudocatenulatum JCM1200^{T}						
	Copy1	Copy2	Copy3	Copy4	Copy5	Copy6
Copy1	100	100	100	100	100	100
Copy2	100	100	100	100	100	100
Copy3	100	100	100	100	100	100
Copy4	100	100	100	100	100	100
Copy5	100	100	100	100	100	100
Copy6	100	100	100	100	100	100
